# Obtención de medios condicionados de células madre mesenquimatosas del estroma de la gelatina de Wharton en un sistema de cultivo dinámico

**DOI:** 10.7705/biomedica.7485

**Published:** 2023-03-02

**Authors:** Juan David Caicedo, Luisa Fernanda Martínez, Elga Johana Vargas, Ítali Segrera, Orlando Chaparro

**Affiliations:** 1 Facultad de Medicina, Universidad Nacional de Colombia, Bogotá, D. C., Colombia Universidad Nacional de Colombia Facultad de Medicina Universidad Nacional de Colombia Bogotá D. C. Colombia

**Keywords:** células madre mesenquimatosas, medicina regenerativa, medios de cultivo condicionados, Mesenchymal stromal stem cells, regenerative medicine, culture media, conditioned

## Abstract

**Introducción.:**

Las células madre mesenquimatosas del estroma tienen un gran potencial terapéutico por los efectos antiinflamatorios, antiapoptóticos y proangiogénicos que se pueden obtener a partir de su secretoma. La gelatina de Wharton constituye una de las mejores fuentes de células madre mesenquimatosas. El secretoma de estas células contiene moléculas bioactivas capaces de mantener la homeostasis celular y tisular para el tratamiento de enfermedades asociadas con procesos inflamatorios, heridas cutáneas, trastornos neurodegenerativos y diabetes.

**Objetivo.:**

Describir un protocolo para obtener medios condicionados de células madre mesenquimatosas del estroma aisladas de la gelatina de Wharton, mediante cultivos celulares dinámicos, considerando que los sistemas tridimensionales ofrecen ventajas sobre los bidimensionales, al replicar mejor las condiciones del microambiente celular *in vivo.*

**Materiales y métodos.:**

Se utilizaron células madre mesenquimatosas del estroma de la gelatina de Wharton. Se caracterizaron mediante la evaluación de su inmunofenotipo por citometría de flujo y por la multipotencialidad por inducción de la diferenciación a linajes mesenquimatosos *in vitro.* Las células se cultivaron en cultivos bidimensionales (monocapa) y tridimensionales en un biorreactor Applikon Biotechnology de 3L, utilizando microperlas plásticas como microtransportadores. Se estandarizó la obtención de medios condicionados y se detectaron 43 factores angiogénicos, utilizando un sistema de micromatriz de anticuerpos para angiogénesis humana.

**Resultados.:**

Las células aisladas presentaron características típicas de las células madre mesenquimatosas del estroma. El cultivo tridimensional facilitó la escalabilidad y mejoró la secreción de factores angiogénicos, en comparación con el bidimensional.

**Conclusiones.:**

Se utilizaron cultivos tridimensionales y bidimensionales para obtener el secretoma. Los primeros permiten obtener volúmenes significativamente mayores de medio condicionado con aumento de la potencial capacidad angiogénica.

## Abstract (en)

**Introduction.:**

Mesenchymal stem cells have great therapeutic potential due to their anti-inflammatory, anti-apoptotic and pro-angiogenic effects through their secretome. Wharton's gelatin is one of the best sources of mesenchymal stromal stem cells; their secretome is rich in bioactive molecules with the ability to maintain cellular and tissue homeostasis for the treatment of pathologies such as inflammation, skin wounds, neurodegenerative disorders, and diabetes.

**Objective.:**

To describe a protocol for obtaining conditioned media from mesenchymal stromal stem cells isolated from Wharton's jelly through dynamic cell cultures, considering that 3D systems offers advantages over 2D by better replicating the conditions of the cellular microenvironment *in vivo.*

**Materials and methods.:**

The mesenchymal stromal stem cells obtained were characterized *in vitro:* their immunophenotype by flow cytometry and their multipotency by induction of differentiation to mesenchymal lineages. The stem cells were grown in 2D monolayer cultures and 3D cultures, in a 3L Applikon Biotechnology bioreactor using plastic microbeads as microcarriers. Obtention of the conditioned media was standardized and 43 angiogenic factors were detected, using an antibody microarray system for human angiogenesis.

**Results.:**

Isolated cells showed typical characteristics of mesenchymal stromal stem cells. 3D cultures facilitated scalability and improved secretion of angiogenic factors compared to 2D cultures.

**Conclusions.:**

The secretome was obtained with 2D and 3D cultures. Significantly larger volumes of conditioned medium with a potentially increased angiogenic capacity were obtained with 3D cultures.

La medicina regenerativa ha tenido un gran desarrollo en los últimos años, como lo refleja el número de estudios clínicos que se llevan a cabo actualmente, utilizando diversos tipos de células madre, especialmente las mesenquimatosas del estroma. Así, por ejemplo, en la página de estudios clínicos de los *National Institutes of Health* (NIH), a la fecha (enero de 2026), se reportan cerca de 1500 estudios sobre este tipo de células.

Las células madre mesenquimatosas, también denominadas del estroma, son células madre adultas, con gran capacidad de autorrenovación y diferenciación respecto a diversos linajes celulares, particularmente, con respecto a las células originadas en el mesénquima. Este tipo de células han demostrado un gran potencial para la terapia celular ^1^^,^^2^.

El uso de las células madre mesenquimatosas en la terapia celular se basó inicialmente en su potencial de diferenciación. Como estas células tienen la capacidad de migrar hasta el sitio de una lesión como reacción a señales proinflamatorias -lo cual se llama migración dirigida *(homing)-* se postuló inicialmente que podrían proliferar, colonizar el sitio lesionado y diferenciarse al tipo celular apropiado.

Sin embargo, en los trabajos recientes se sugiere un papel más complejo en la función de la regeneración tisular. En los estudios con modelos animales, se ha demostrado que las células mesenquimatosas del estroma:

1. tienen una vida relativamente corta después de la migración dirigida,
2. no se injertan ni se diferencian para formar nuevos tejidos y
3. ejercen su efecto terapéutico, principalmente, mediante factores que secretan al microambiente celular.

Entre estos, se han identificado factores de crecimiento, como interleucinas, citocinas y proteasas, los cuales ejercen acciones antiinflamatorias, antiapoptóticas y proangiogénicas; además, promueven la proliferación celular y el reclutamiento de células madre, lo que se conoce como efecto paracrino ^3^^,^^4^.

El conjunto de todos los factores y moléculas secretadas por las células mesenquimatosas del estroma se conoce como secretoma, y puede recolectarse del sobrenadante de los medios de cultivo de estas células. Estos medios de cultivo se denominan medios condicionados ^5^.

Estos medios condicionados han demostrado su potencial terapéutico en el tratamiento de enfermedades isquémicas -tanto agudas como crónicas, como el infarto del miocardio y el accidente cerebrovascular-, de enfermedades neurodegenerativas y de lesiones de la médula espinal ^6^^-^^8^; asimismo, para alopecia, heridas crónicas, lesión o insuficiencia hepática aguda, lesión pulmonar y enfermedad periodontal, entre otras ^9^^,^^10^.

El cordón umbilical es un tejido perinatal relativamente fácil de obtener y es una fuente prometedora de células madre. Las células madre mesenquimatosas del estroma derivadas del cordón umbilical, se consideran una de las principales fuentes de este tipo de células para aplicaciones terapéuticas ^11^. Se han aislado de diferentes componentes del cordón umbilical, como la gelatina de Wharton que es el tejido conjuntivo que rodea sus vasos. Por tratarse de una población celular primitiva del estroma, las células madre mesenquimatosas aisladas de la gelatina de Wharton ofrecen la ventaja de tener una mayor tasa de proliferación y una menor inmunogenicidad, en comparación con las obtenidas de tejido adulto ^12^. Entre los distintos componentes del cordón umbilical, la gelatina de Wharton se ha descrito como la mejor fuente de células madre mesenquimatosas del estroma ^13^.

El secretoma de las células madre aisladas de la gelatina de Wharton es rico en moléculas bioactivas con capacidad de mantener la homeostasis celular y tisular ^14^. Dadas estas características, se han publicado varios estudios en los que se usa el secretoma para tratar la inflamación, las heridas de piel, los trastornos neurodegenerativos, la fibrosis tisular y la diabetes ^12^. Vale la pena resaltar, por lo tanto, el gran potencial del secretoma de estas células en la medicina regenerativa y en la terapia acelular. Sin embargo, se requieren estudios más detallados sobre su perfil proteómico y metabolómico, para comprender mejor sus componentes y mecanismos de acción, celulares y moleculares ^15^.

Tradicionalmente, las células madre mesenquimatosas se han cultivado en monocapa, según las técnicas estándar de cultivos bidimensionales, los cuales presentan varias limitaciones y riesgos. En primer lugar, son muy laboriosas y costosas en términos de mano de obra y tiempo de trabajo. En segundo lugar, y tal vez lo más importante, es que no reproducen de manera eficiente las condiciones de su nicho celular *in vivo,* por lo cual las células se someten a unas condiciones artificiales de adhesión, configuración bidimensional, movilidad y comunicación celular.

En condiciones fisiológicas *in vivo,* estas células madre están embebidas en la matriz extracelular en una configuración tridimensional, en la que se producen interacciones entre células, y entre las células y la matriz. Por lo tanto, los cultivos en monocapa representan un ambiente artificial de adhesión, configuración bidimensional y movilidad celular.

Está demostrado que la expansión celular en una estructura bidimensional disminuye la capacidad de proliferación, diferenciación y formación de colonias, e induce senescencia después de varias transferencias del cultivo ^16^^,^^17^. Por lo tanto, es necesario generar sistemas de cultivo que permitan a las células madre mesenquimatosas la interacción tridimensional, para que los estudios *in vitro* repliquen de manera más fiel las condiciones *in vivo*^17^.

Desde este punto de vista, los sistemas de biorreactores representan una de las soluciones más viables para la expansión a gran escala de las células madre mesenquimatosas. Se han utilizado diferentes sistemas de biorreactores para este propósito: biorreactores de lecho fijo, de suspensión con agitación, de perfusión y de fibra hueca, entre otros ^18^. Uno de los más utilizados es el biorreactor de suspensión con agitación con microtransportadores. Este sistema facilita la obtención de una gran densidad celular ^16^^,^^18^.

En el presente trabajo, se describen los pasos para aislar células madre mesenquimatosas del estroma de la gelatina de Wharton y para obtenerlas de medios condicionados en un sistema de cultivo tridimensional, utilizando un biorreactor de suspensión con agitación acoplado a microtransportadores.

## Materiales y métodos

### Aislamiento y caracterización

Para aislar las células se utilizó el método de explantes, Se recolectaron cordones umbilicales frescos, de partos a término por cesárea, provenientes de donantes sanas de la sala de partos de la Clínica de la Mujer de Bogotá, previo diligenciamiento del consentimiento informado por parte de la madre. Luego, los cordones se transportaron al laboratorio, debidamente refrigerados e inmersos en una solución salina tampón *(Phosphate Buffered Saline,* PBS) (PBS 1X, GIBCO), con suplemento al 1 % de antibiótico y antimicótico (Sigma Aldrich Inc.). Todos los pasos del lavado, la obtención de la gelatina de Wharton y la siembra de los explantes, se hicieron siguiendo los protocolos descritos por Goyal *et al.*^19^.

Se sembraron explantes de 0,2 cm de diámetro, aproximadamente, en cajas plásticas de cultivo de seis pozos (Corning, Inc.), en medio Eagle modificado por Dulbecco bajo en glucosa (DMEM, GIBCO), suplementado con suero fetal bovino al 10 % (SFB^TM^, GIBCO), y 100 U/ml de penicilina y 100 μg/ml de estreptomicina (Sigma Aldrich Inc.).

Los explantes se incubaron a 37 °C en atmósfera húmeda con 5 % de CO_2_. El medio de cultivo fue remplazado por un medio fresco dos veces por semana hasta que las células alcanzaron del 70 al 80 % de confluencia, momento en el cual se retiraron los explantes.

Para los subcultivos celulares, las células madre aisladas de la gelatina de Wharton se desprendieron utilizando la enzima TrypLE™ Select (Life Technologies, Thermo-Fisher Scientific), se lavaron con solución tampón PBS y se sembraron en frascos de cultivo de 75 cm^2^, con una densidad de siembra de 5000 células por cm^2^.

Las células madre se caracterizaron de acuerdo con los criterios establecidos por la *International Society for Cellular Therapy*^20^. El inmunofenotipo se evaluó mediante citometría de flujo, identificando los marcadores de superficie CD105, CD73, CD90, CD34 y CD45. La multipotencialidad se evaluó mediante inducción de la diferenciación *in vitro* a linajes osteogénico, condrogénico y adipogénico, como lo describen Linero *et al.*^21^.

### Ensayos de adhesión celular

Para el cultivo de las células madre mesenquimatosas aisladas de la gelatina de Wharton en condiciones tridimensionales en el biorreactor, se utilizaron como microtransportadores las perlas SoloHill™ Plastic Plus Microcarriers (Sartorius, Cat No. PPIR-221-020). La adhesión celular a los microtransportadores se evaluó utilizando una densidad celular de 3x10^4^ células por cm^2^ y siguiendo las recomendaciones del fabricante ^18^.

### Cultivos 3D y obtención de los medios condicionados

Para los cultivos 3D o tridimensionales se utilizó el Sistema Minibio de 3L con controlador My-control™ (Applikon Biotechnology, z4mmy0002c).

El biorreactor se configuró siguiendo las instrucciones del fabricante. Se verificó que los efectores (rotor de agitación, bomba de NaHCO_3_, válvula de aire y válvula de entrada de CO_2_) estuvieran funcionando correctamente y se calibraron los sensores del equipo (termómetro, sensor de O_2_ y sensor de pH) utilizando los estándares respectivos.

Los microtransportadores se esterilizaron en autoclave, a 121 °C, en agua desionizada, siguiendo las instrucciones del fabricante. Las células madre mesenquimatosas aisladas de la gelatina de Wharton (2 x 10^6^ células) se resuspendieron en 20 ml de medio de cultivo fresco y se agregaron los microtransportadores Plastic Plus™ (1,5 g por litro). La mezcla se incubó durante 40 minutos para permitir la adhesión de las células a los microtransportadores. Previamente, se habían estabilizado las condiciones en el biorreactor, incubando 750 ml de medio completo fresco (medio de Eagle modificado por Dulbecco bajo en glucosa más suero fetal bovino al 10 %). Se ajustaron las condiciones de pH, temperatura y agitación, según los valores óptimos recomendados (pH 7,2 a 8,5, temperatura 37 °C y agitación inicial 60 revoluciones por minuto). Se monitorizaron continuamente los sensores del equipo para asegurarse de que las condiciones se mantuvieran dentro de los rangos deseados.

Por último, se transfirieron las células adheridas a los microtransportadores y se ajustó el volumen final a 1 litro.

El cultivo se mantuvo durante tres días, asegurando la monitorización periódica de los sensores, y se ajustaron los parámetros del biorreactor según lo necesario para mantener las condiciones óptimas. Al cuarto día, se detuvo el sistema y se reemplazó la mitad del medio de cultivo con medio fresco (medio de Eagle modificado por Dulbecco bajo en glucosa más suero fetal bovino al 10 %). Se reactivó el sistema del biorreactor y se dejó en incubación tres días más.

Para la obtención de los medios condicionados, se detuvo el funcionamiento del biorreactor, las células adheridas a los microtransportadores se decantaron y se retiró el medio de cultivo. Las células se lavaron con solución tampón PBS y se agregó 1 L de medio de Eagle modificado por Dulbecco bajo en glucosa y libre de proteínas. Se restablecieron las condiciones de cultivo en el biorreactor y se mantuvo por 48 horas más, al cabo de las cuales se recuperó el medio condicionado, el cual se fraccionó en alícuotas y se almacenó a -20 °C.

### Análisis de factores angiogénicos-arreglo de anticuerpos

Se obtuvieron 130 mg de proteína total de cada medio condicionado de las células madre mesenquimatosas aisladas de la gelatina de Wharton cultivadas, tanto en monocapa (2D) como en el biorreactor (3D). Estas proteínas se analizaron de manera comparativa y se detectaron 43 proteínas humanas, incluidas citocinas, factores de crecimiento, proteasas y receptores solubles, utilizando el kit de arreglo de anticuerpos para angiogénesis humana de Ray Biotech™ (RayBio Human Angiogenesis Antibody Array C Series 1000), siguiendo las instrucciones del fabricante.

Para el revelado, se utilizó una película de rayos X (G Kodak™ X-ray Film 01) la cual se expuso por 60 segundos. La intensidad de la señal obtenida para cada factor se cuantificó mediante el *software* Imagen J1.4 de los NIH. Las densidades de la señal (pixeles) se normalizaron por miligramo de proteína y los valores se expresaron como unidades arbitrarias por miligramo de proteína.

### Consideraciones éticas

Este estudio fue aprobado por el Comité de Ética de Investigación en Salud de la Clínica de la Mujer, con fecha del 18 de enero de 2019. Se cuenta con el consentimiento informado de todas las personas que participaron en el estudio y se respetó su derecho a la privacidad.

## Resultados

Aislamiento y caracterización de las células madre mesenquimatosas del estroma aisladas de la gelatina de Wharton

Las células madre mesenquimatosas se han aislado de diferentes componentes del cordón umbilical -el cual se descarta después del nacimiento- por ser una fuente de células madre de fácil acceso y sin las limitaciones éticas de otras fuentes. El método de explante utilizado en este estudio permitió el aislamiento de células madre mesenquimatosas, que tenían las características fenotípicas previamente descritas: morfología fibroblastoide y adhesión al plato de cultivo ^20^.

**Figura 1:**
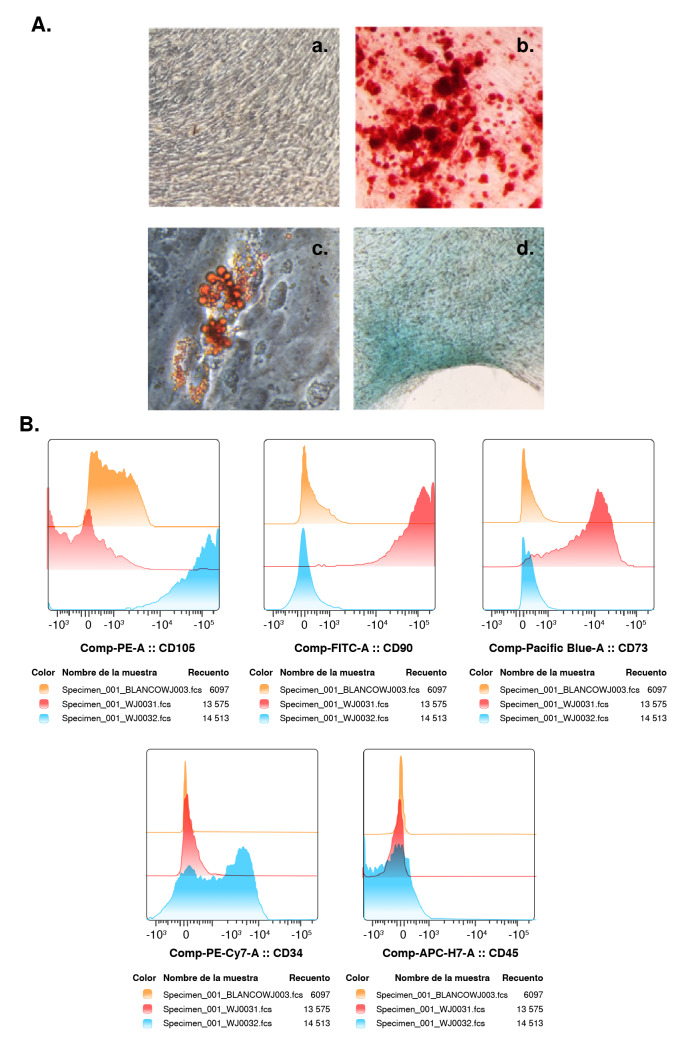
Caracterización *in vitro* de las células madre mesenquimatosas del estroma aisladas de la gelatina de Wharton. **A.** Diferenciación *in vitro:* a. Control; b. Diferenciación osteogénica; c. Diferenciación adipogénica; d. Diferenciación condrogénica. **B.** Inmunotipificación: células positivas para CD105, CD90 y CD73, y negativas para CD34 y CD45.

La multipotencialidad de estas células se demostró por su capacidad de diferenciarse *in vitro* en linajes osteogénicos, adipogénicos y condrogénicos (figura 1A). La caracterización por citometría de flujo mostró que las células aisladas expresaban los marcadores de superficie particulares de este tipo celular: positivas para CD105 (96,4 %), CD73 (94,8 %) y CD90 (97,1 %), y negativas para CD34 (9,59 %) y CD45 (2,85 %) (figura 1B).

Por otra parte, el método de explante demostró ser muy eficiente e hizo innecesario un tratamiento enzimático, lo que reduce la posibilidad de contaminación por endotoxinas y lo hace más económico ^19^.

### Cultivos tridimensionales y obtención de los medios condicionados

El primer paso para el establecimiento de los cultivos 3D o tridimensionales en el biorreactor, es comprobar la adherencia de las células madre mesenquimatosas a los microtransportadores. Entre las opciones que se analizaron, las perlas SoloHill Plastic Plus Microcarriers™ mostraron las características más favorables. Además de la facilidad y conveniencia para su preparación y manejo, se alcanzó una adhesión celular superior al 85 %.

### Análisis de factores angiogénicos-arreglo de anticuerpos

Usando la micromatriz de anticuerpos para angiogénesis humana C1000 (RayBiotech, Norcross, GA, USA), se demostró que tanto las células madre mesenquimatosas cultivadas en monocapa (2D) como las cultivadas en el biorreactor (3D), secretan 43 factores angiogénicos (citocinas, factores de crecimiento, proteasas y receptores solubles) y que el patrón de secreción es muy similar en las dos condiciones de cultivo.

**Figura 2:**
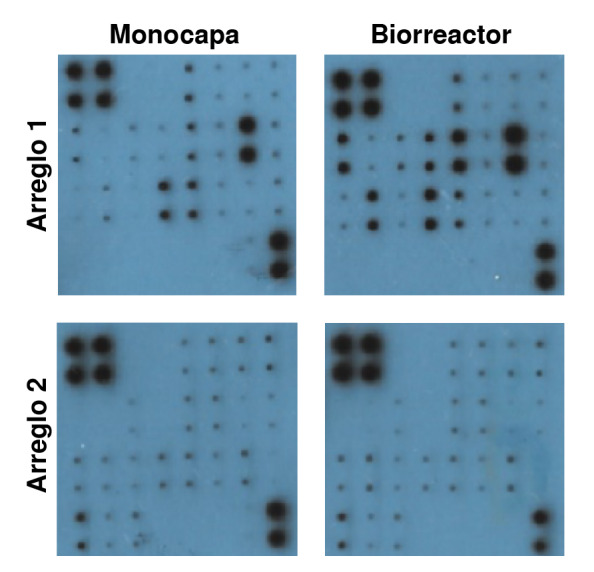
Micromatriz de anticuerpos C1000 (RayBiotech, Norcross, GA, USA) para angiogénesis humana

Se observó un incremento significativo en la secreción de algunos factores en los cultivos tridimensionales, como la leptina, y las interleucinas IL 6 y IL8, MCP1, RANTES, TIMP-1 y angiopoyetina 1, lo que sugiere un aumento de la capacidad angiogénica de los medios condicionados obtenidos en cultivo 3D (figura 2, cuadro 1).

**Cuadro 1:** Comparación de los niveles aumentados de secreción de los factores de crecimiento y citocinas en los cultivos 3D vs los 2D de las células madre mesenquimatosas del estroma aisladas de la gelatina de Wharton

Factor/citocina	Monocapa	Biorreactor
	(UA/mg proteína)	(UA/mg proteína)
Leptina	0,299	1,028
IL 6	0,033	2,150
IL 8	1,078	4,783
MCP1	6,566	8,795
RANTES	0,313	2,654
TIMP1	2,429	3,416
Angiopoyetina 1	0,296	0,445

UA: unidades arbitrarias; MCP1: *monocyte chemoattractant protein-1;* RANTES: *regulated on activation, normal T cell expressed and secreted* TIMP: *tissue inhibitor of metalloproteinases*

## Discusión

La medicina regenerativa es uno de los campos de más rápido crecimiento en el área de las ciencias biomédicas y es considerada como "la medicina del futuro". Mediante la terapia celular, su principal herramienta, se enfoca en la regeneración de células, tejidos u órganos lesionados. Las células madre mesenquimatosas, en particular, se han constituido en uno de sus pilares fundamentales, dadas sus características de baja inmunogenicidad y gran capacidad de proliferación y diferenciación.

Las propiedades únicas de las células madre mesenquimatosas del estroma aisladas de la gelatina de Wharton han llamado la atención de la comunidad científica desde hace varios años ^22^. El hecho de que puedan aislarse de una manera relativamente fácil y eficiente a partir de un tejido abundante -y que normalmente se desecha-, en un procedimiento que no implica ningún riesgo ni molestia ni para la madre ni para el recién nacido y, además, sin cuestionamientos éticos, las convierte en una fuente muy llamativa de células madre mesenquimatosas en el campo de la investigación y de la aplicación clínica ^11^^,^^15^.

Las células aisladas de la gelatina de Wharton en nuestro laboratorio cumplen con los criterios mínimos establecidos por la *International Society for Cellular Therapy* para caracterizar a las células madre mesenquimatosas ^20^: crecen adheridas a la superficie del plato de cultivo con una morfología fibroblastoide (figura 1), presentan un inmunofenotipo característico y poseen la capacidad de diferenciación multipotencial. La técnica de explantes para aislarlas, además de ser muy eficiente, permite aislar poblaciones más puras, sin usar enzimas de origen animal, lo cual reduce los riesgos de contaminación ^23^.

Los resultados de las investigaciones de los últimos años sugieren que el principal efecto terapéutico de las células madre mesenquimatosas se ejerce mediante su actividad paracrina al secretar al medio extracelular una serie de moléculas o factores, directamente o en vesículas extracelulares (exosomas y microvesículas). El conjunto de estos diferentes tipos de moléculas y factores bioactivos se conoce como secretoma, e incluye citocinas proinflamatorias y antiinflamatorias, quimiocinas, antiapoptóticos, proangiogénicos y antiangiogénicos, y neuroprotectores, entre otros. El secretoma es el principal responsable del mecanismo de acción de las células madre mesenquimatosas en los procesos de regeneración tisular.

Los factores que componen el secretoma se pueden recolectar en los medios condicionados de las células madre mesenquimatosas y, actualmente, se consideran una alternativa al uso de las células en la terapia celular, en lo que se ha denominado "terapia libre de células".

Los resultados del presente trabajo muestran que el sistema de cultivo tridimensional de las células madre mesenquimatosas aisladas de la gelatina de Wharton y permite aumentar su producción a partir de los medios condicionados, en un volumen significativamente mayor en comparación con el cultivo en monocapa.

Por otra parte, al analizar el efecto angiogénico del secretoma como un parámetro indicador, se encontró que, aunque los secretomas recolectados en las dos condiciones de cultivo (2D y 3D) tienen una composición muy similar, el aumento de un grupo de los factores angiogénicos en los medios condicionados recolectados en el biorreactor sugiere una mayor capacidad angiogénica. Estos resultados deberán ser corroborados en estudios funcionales posteriores.

Los resultados obtenidos coinciden con los de otras investigaciones disponibles en la literatura, en cuanto a que destacan las ventajas de los cultivos 3D frente a los 2D para mejorar las propiedades terapéuticas del secretoma derivado de las células madre mesenquimatosas.

En el presente estudio se encontró que el cultivo tridimensional de células madre mesenquimatosas aisladas de la gelatina de Wharton permite obtener un medio condicionado de mayor volumen y con una capacidad angiogénica superior, lo cual coincide con otros estudios que informan mayor funcionalidad mediante sistemas 3D dinámicos. Por ejemplo, los medios condicionados obtenidos en sistemas 3D dinámicos con células madre mesenquimatosas derivadas de adipocitos, conservaron su potencial angiogénico y demostraron eficacia en modelos *in vitro* e *in vivo,* incluyendo la cicatrización de heridas ^24^. Además, la hipoxia en cultivos 3D incrementó el efecto terapéutico, lo que subraya la importancia de replicar el microambiente celular *in vivo* para maximizar los beneficios del secretoma.

Por otra parte, en los estudios con células madre mesenquimatosas modificadas genéticamente para sobreexpresar proteínas específicas -como la osteonectina, SPARC *(Secreted Protein Acidic and Rich in Cysteine)-* se resalta que estas modificaciones pueden potenciar aún más las propiedades regenerativas del secretoma, lo que plantea una posible sinergia entre el uso de cultivos 3D y técnicas de bioingeniería ^25^. Además, los investigadores coinciden en que los cultivos 3D son eficientes para producir a mayor escala el secretoma, ahorran tiempo y espacio, y mejoran su perfil terapéutico. Sin embargo, en todos los estudios se destaca la necesidad de enfrentar desafíos como la heterogeneidad biológica de las células madre mesenquimatosas del estroma y la estandarización de los protocolos de cultivo; ambos son aspectos clave para garantizar la reproducibilidad, y la aplicación clínica segura y efectiva de estos tratamientos ^26^.

Dado que el patrón de secreción de las células madre mesenquimatosas puede variar según el microambiente en que se reproducen, los estímulos externos, cada vez se abre más la posibilidad de preacondicionar las células para inducir la producción de un secretoma con propiedades particulares, que pueda usarse para tratar enfermedades específicas. Así, por ejemplo, se ha reportado el preacondicionamiento de células madre mesenquimatosas para inducir un secretoma con mejores capacidades inmunomoduladoras ^25^^-^^27^, o con una mayor capacidad de inducir la cicatrización de heridas cutáneas o la regeneración de cartílago articular ^25^^,^^28^.

El secretoma permite reducir significativamente los riesgos potenciales del trasplante celular y mantener su capacidad terapéutica. Además, cuenta con una serie de ventajas adicionales -como la posibilidad de escalar su producción, su fraccionamiento y concentración, la liofilización, y las facilidades de almacenamiento y distribución-, lo cual redunda en su mayor accesibilidad y reduce significativamente los costos, tanto para los pacientes como para los sistemas de salud. Por lo tanto, la aplicación de "terapias libres de células" basadas en los componentes secretados por las células madre mesenquimatosas como una nueva clase de productos biofarmacéuticos, representa un enfoque prometedor y de rápido desarrollo en la medicina regenerativa ^29^^,^^30^.
